# Photoluminescence of Homoleptic Lanthanide Complexes With Tris(benzotriazol-1-yl)borate

**DOI:** 10.1007/s10895-021-02772-7

**Published:** 2021-07-13

**Authors:** Marco Bortoluzzi, Valentina Ferraro, Federica Sartor

**Affiliations:** Dipartimento di Scienze Molecolari e Nanosistemi, Università Ca’ Foscari Venezia, Via Torino 155, 30172 Mestre, VE Italy

**Keywords:** Lanthanides, Scorpionates, Ln^3+^ transitions, Benzotriazole, Doped polymers

## Abstract

Bright photoluminescent neutral complexes having general formula [Ln(tbtz)_3_] (Ln = Eu, Tb; tbtz = tris(benzotriazol-1-yl)borate) were obtained by reacting K[tbtz] with EuCl_3_ and TbCl_3_. The emissions in the visible range, related to the f-f transitions of the trivalent lanthanide ions, are observable upon excitation with wavelengths shorter than 350 nm. The most intense emission bands correspond to the ^5^D_0_ → ^7^F_4_ transition at 699 nm for the europium complex and to the ^5^D_4_ → ^7^F_5_ transition at 542 nm for the terbium derivative. The luminescence is in all the cases mostly associated with the antenna-effect from the coordinated tbtz ligands. The synthetic approach was successfully extended to the preparation of the analogous yttrium and gadolinium derivatives. Tricapped trigonal prismatic geometry was attributed to the complexes on the basis of luminescence data and DFT calculations. Highly photoluminescent plastic materials were obtained by embedding small amounts of [Eu(tbtz)_3_] or [Tb(tbtz)_3_] in poly(methyl methacrylate).

## Introduction

Luminescent complexes of trivalent lanthanide ions are of widespread interest for advanced technologies [1–5]. Selected examples of application include electronic devices [6, 7], electroluminescent materials [8, 9], photovoltaics [10], biological probes and multifunctional materials [11–20].

Poly(pyrazol-1-yl)borate ligands were widely employed for the preparation of lanthanide complexes, that were investigated for their structural, magnetic and luminescent features [21–32]. In particular, coordination compounds having the general formula [Ln(Tp)_3_], where Tp is tris(pyrazol-1-yl)borate, were characterized by means of single crystal X-ray diffraction. It was observed that the metal centre in homoleptic tris(pyrazol-1-yl)borate lanthanide complexes can be nine- or eight-coordinated depending upon its ionic radius. The electronic structures of the lanthanide centres were investigated in detail and antenna-effect was detected for some visible- and NIR-emitting complexes [33–39]. The pure complexes showed high thermal stability [39]. Appreciable photoluminescence was maintained also after dispersion in acrylic-based polymers, or after mixing the complexes with different inorganic pigments and suitable binders [40, 41].

The functionalization of the poly(pyrazol-1-yl)borate skeleton demonstrated to be a viable route for the preparation of new luminescent lanthanide complexes. For instance, the coordination of bis[3-(2-pyridyl)pyrazol-1-yl]dihydroborate to Yb(III), Nd(III) and Er(III) afforded NIR-emitting species. The analogous Pr(III) derivative showed luminescence both in the NIR and in the visible regions [42].

Another approach to prepare new scorpionate-based luminescent compounds is the replacement of the pyrazol-1-yl moieties with azoles characterized by different donor ability and steric bulk, together with improved light-harvesting properties [43]. In this paper we describe the straightforward preparation of new homoleptic lanthanide complexes with tris(benzotriazol-1-yl)borate in the coordination sphere. Particular attention was devoted to the photophysical characterization of the europium and terbium complexes, that allowed to highlight analogies and differences with the homoleptic tris(pyrazol-1-yl)borate complexes already reported. Finally, the new compounds were studied as dopants for the preparation of photoluminescent polymers.

## Experimental Section

### Chemicals

Commercial organic solvents (toluene ≥ 99.5%, diethyl ether ≥ 99.7%, dichloromethane ≥ 99.8%) were purchased from Sigma Aldrich (Germany) and purified with Na/benzophenone (toluene, diethyl ether) or CaH_2_ (dichloromethane) following literature methods [44]. 1*H*-benzotriazole (≥ 99.0%) and potassium borohydride (98%) were Sigma Aldrich (Germany) products, used as received. Yttrium and lanthanide chlorides (99.9% in all the cases) were Strem Chemicals (France) products, used without further purifications. Poly(methyl methacrylate) (PMMA, M_w_ = 86,000 g mol^−1^) was purchased from TCI Chemicals (Belgium) and used as received. Potassium tris(benzotryazol-1-yl)borate, K[tbtz], was synthesized accordingly to literature procedures [45–47].

### Characterization Methods

Elemental analyses (C, H, N) were carried out using an Elementar micro elemental analyzer, model UNICUBE® (Germany). Magnetic susceptibilities were measured on solid samples at 298 K with a Sherwood Scientific magnetic susceptibility balance, model MK1 (UK), and corrected for diamagnetic contribution by means of tabulated Pascal’s constants [48]. Conductivity measurements were carried out using a Radiometer Copenhagen instrument, model CDM83 (Denmark). IR spectra were collected in the 4000 − 400 cm^−1^ range using a Perkin-Elmer spectrophotometer, model Spectrum One (UK). ^1^H NMR spectra were recorded with a Bruker instrument operating at 300.13 MHz, model Avance 300 (Germany). The spectra were recorded in deuterated dimethylsulfoxide (99.90% D, Eurisotop, France), used as received. The ^1^H NMR chemical shifts were referred to the partially deuterated fraction of the solvent (2.50 ppm), itself quoted with respect to tetramethylsilane (δ = 0.00 ppm).

Absorption spectra in dichloromethane solution were collected using a Perkin-Elmer spectrophotometer, model Lambda 35 (UK). Photoluminescence emission (PL) and excitation (PLE) measurements were carried out at room temperature on solid samples by a Horiba Jobin Yvon spectrofluorometer, model Fluorolog-3 (France). A continuous-wave xenon arc lamp was used as source selecting the excitation wavelength by a double Czerny-Turner monochromator. A single grating monochromator coupled to a Hamamatsu Photonics R928 photomultiplier tube (Japan) was used as detection system for optical emission measurements. Excitation and emission spectra were corrected for the instrumental functions. Time-resolved analyses were performed in Multi Channel Scaling modality (MCS, 3000 channels, 5000 ns/channel) by using a pulsed Ekspla NT/342 Nd:YAG laser (Lithuania) equipped with an optical parametric oscillator. The pulse duration of the laser was 6 ns and the repetition rate was 10 Hz. The channels related to the time before and during the excitation pulse were removed from the subsequent analysis.

### Synthesis of [Ln(tbtz)_3_] Complexes (Ln = Y, Eu, Gd, Tb) and Doped Polymers

YCl_3_ or LnCl_3_ (Ln = Eu, Gd, Tb) (5.0 mmol) was dissolved in 10 mL of distilled water. 5 mL of 0.1 M HCl was then added. A solution containing 1.5 mmol (0.608 g) of K[tbtz] in 20 mL of H_2_O was slowly added to the reaction mixture. After 24 h under stirring at room temperature the white solid formed was collected by filtration, washed with 5 mL of water and dried under vacuum in the presence of P_4_O_10_. The product was purified by dissolution in hot toluene (100 mL), and the solution thus obtained was quickly filtered to remove the residual particulate. The solvent was then removed under reduced pressure. The addition of diethyl ether (20 mL) caused the separation of a white solid, that was collected by filtration, washed with 5 mL of diethyl ether and dried under vacuum. Yield ≥ 70% in all the cases.

Doped polymers, generally indicated as [Ln(tbtz)_3_]@PMMA, were prepared from dichloromethane solutions of the complexes and commercial PMMA. In a typical preparation, a weighed amount of [Ln(tbtz)_3_] (from 0.020 to 0.060 g) was dissolved in 5 mL of dichloromethane. The solution was added to a gently stirred solution of PMMA (1.000 g) in 40 mL of CH_2_Cl_2_. The solution was then concentrated at a reduced pressure to about 3 mL and transferred into a round polyethylene holder having 1 cm diameter. The residual solvent was allowed to evaporate in air at room temperature. The final plastic material was kept under vacuum overnight to remove the traces of solvent.

### Characterization of [Ln(tbtz)_3_] Complexes

**Ln = Y**. Anal. calcd for C_54_H_39_B_3_N_27_Y (1187.41 g mol^−1^, %): C, 54.62; H, 3.31; N, 31.85. Found (%): C, 54.39; H, 3.33; N, 31.72. IR (cm^−1^): 2426 (ν_BH_). ^1^H NMR (DMSO-d_6_, 298 K) δ: 7.78 (2 dd, 2H, ^3^*J*_HH_ = 7.1 Hz, ^4^*J*_HH_ = 1.3 Hz, benzotriazole-H5 and benzotriazole-H6); 7.26, 7.13 (2 td, 2H, ^3^*J*_HH_ = 7.1 Hz, ^4^*J*_HH_ = 1.3 Hz, benzotriazole-H4 and benzotriazole-H7).

**Ln = Eu**. Anal. calcd for C_54_H_39_B_3_N_27_Eu (1250.47 g mol^−1^, %): C, 51.87; H, 3.14; N, 30.24. Found (%): C, 51.66; H, 3.18; N, 30.10. χ^M^_corr_ (c.g.s.u., 298 K): 4.85·10^–3^. IR (cm^−1^): 2431 (ν_BH_). ^1^H NMR (DMSO-d_6_, 298 K) δ: 7.80 (2 d, slightly br, 2H, ^3^*J*_HH_ = 6.8 Hz, benzotriazole-H5 and benzotriazole-H6); 7.28, 7.15 (2 t, slightly br, 2H, ^3^*J*_HH_ = 6.8 Hz, benzotriazole-H4 and benzotriazole-H7).

**Ln = Gd**. Anal. calcd for C_54_H_39_B_3_N_27_Gd (1255.75 g mol^−1^, %): C, 51.65; H, 3.13; N, 30.12. Found (%): C, 51.44; H, 3.17; N, 29.98. χ^M^_corr_ (c.g.s.u., 298 K): 2.64·10^–2^. IR (cm^−1^): 2431 (ν_BH_).

**Ln = Tb**. Anal. calcd for C_54_H_39_B_3_N_27_Tb (1257.43 g mol^−1^, %): C, 51.58; H, 3.13; N, 30.08. Found (%): C, 51.37; H, 3.18; N, 29.96. χ^M^_corr_ (c.g.s.u., 298 K): 4.03·10^–2^. IR (cm^−1^): 2431 (ν_BH_). ^1^H NMR (DMSO-d_6_, 298 K) δ: 7.82, 7.67, 7.21, 7.08 (4 s, br, 4H, benzotriazole hydrogen atoms).

### Computational Details

The computational geometry optimizations were carried out without symmetry constraints using the hybrid-GGA DFT functional EDF2 [49], in combination with the split-valence polarized 6–31G(d,p) basis set for light atoms and the LANL2DZ basis set for yttrium [50]. The scalar quasirelativistic 4f-in-core pseudopotentials ECP52MWB, ECP53MWB and ECP54MWB were used respectively for europium, gadolinium and terbium, with the associated valence basis sets [51–53]. Because of the inclusion of the 4f^n^ shell of Ln(III) in the pseudopotential, the “restricted” formalism was applied [54]. Calculations were performed with Spartan’16 (Wavefunction Inc., USA), build 2.0.3 [55], running on Intel Xeon-based x86–64 workstations.

## Results and Discussion

**Scheme 1:**
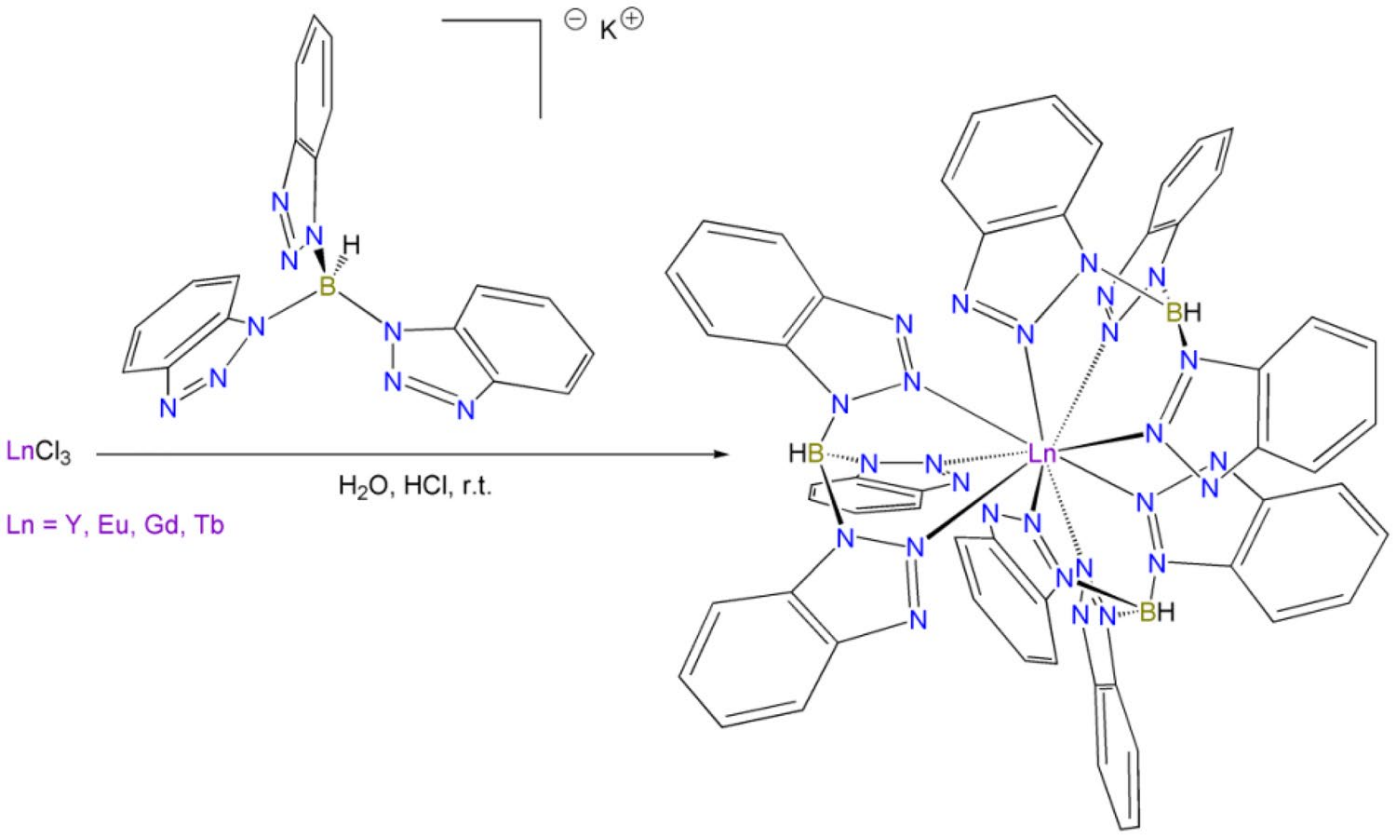
Synthesis of [Ln(tbtz)_3_] complexes

Homoleptic tris(benzotriazol-1-yl) borate lanthanide complexes having general formula [Ln(tbtz)_3_] (Ln = Y, Eu, Tb) were easily synthesized from the reaction of the corresponding metal chlorides and three equivalents of K[tbtz] in slightly acidic water (Scheme 1). They were purified by dissolution in hot toluene, followed by precipitation with diethyl ether. Elemental analysis data are in agreement with the proposed formulations and dichloromethane solutions of the complexes are non-conductive. The experimental magnetic moments at room temperature are 3.3 BM for [Eu(tbtz)_3_] and 9.8 BM for [Tb(tbtz)_3_], in agreement with the values expected for complexes of trivalent europium and terbium ions [56]. The IR spectra are closely comparable and show a band around 2430 cm^−1^ attributable to ν_B-H_ stretching. The ^1^H NMR spectra in DMSO-d_6_ at room temperature are composed by two superimposed doublets and two triplets for the benzotrazol-1-yl groups, indicating that all the coordinating moieties are equivalent on the NMR timescale. The presence of paramagnetic centres causes the expected broadening of the resonances.

**Fig. 1:**
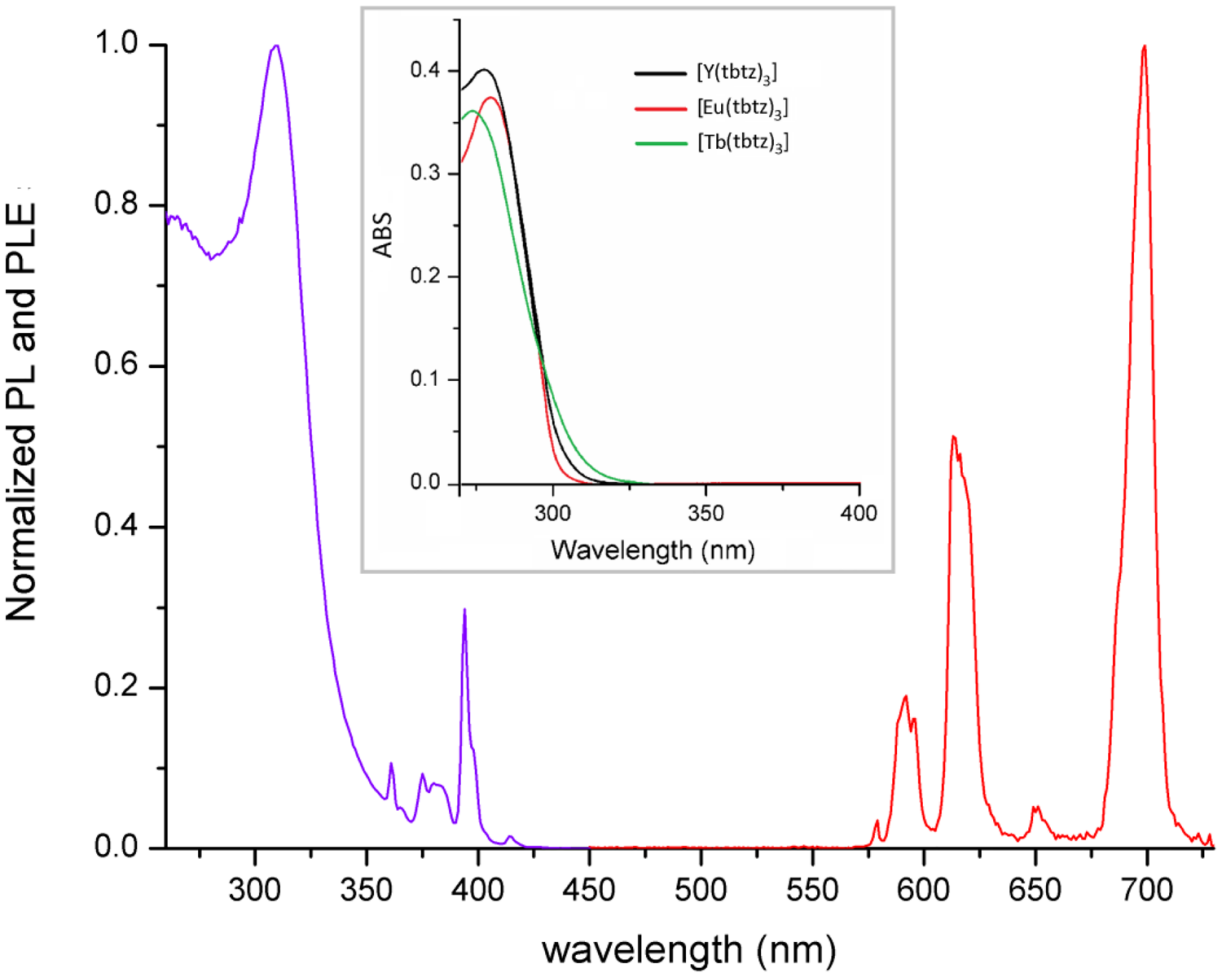
PL (red line, λ_excitation_ = 310 nm) and PLE (violet line, λ_emission_ = 700 nm) of solid [Eu(tbtz)_3_] at room temperature. Inset: absorption spectra of 10^–5^ M CH_2_Cl_2_ solutions of [Ln(tbtz)_3_] complexes

The UV–VIS spectra of the complexes show intense absorptions for wavelengths below 300 nm (Fig. 1), with maxima around 278 nm and molar extinction coefficients close to 40,000 M^−1^ cm^−1^. [Eu(tbtz)_3_] and [Tb(tbtz)_3_] are appreciably photoluminescent under UV irradiation, both as solid samples and in dichloromethane solution. The PL spectrum of [Eu(tbtz)_3_] reported in Fig. 1 is composed by the typical ^5^D_0_ → ^7^F_J_ (J = 0 – 4) transitions of the metal centre between 580 and 700 nm, without any signal attributable to luminescence from the coordinated ligands. The related PLE spectrum (Fig. 1) shows a noticeable antenna-effect for wavelengths below 350 nm, with a maximum at 309 nm. The dominant contribution of coordinated tbtz to the sensitization of Eu(III) luminescence is evidenced by the relative intensity of the ligand-related band with respect to the direct excitations of the metal centre, the ^5^L_6_ ← ^7^F_0_ at 394 nm in particular. The presence of a single ^5^D_0_ → ^7^F_0_ band at 579 nm in the PL spectrum suggests the presence of only one emitting species, even if such information is not conclusive [57]. The relatively low intensity of the ^5^D_0_ → ^7^F_2_ hypersensitive band centred around 616 nm and the quite low I(^5^D_0_ → ^7^F_2_):I(^5^D_0_ → ^7^F_1_) intensity ratio, around 3:1, indicate a symmetric coordination sphere surrounding the metal ion. The most intense band, corresponding to about 60% of the total emission, is the ^5^D_0_ → ^7^F_4_ one, centred at 699 nm. Such a feature is relatively uncommon and it was observed for instance for the tris(pyrazol-1-yl)borate complex [Eu(Tp)_3_] [39]. The relative intensities of the transitions in the PL spectrum suggest that [Eu(tbtz)_3_] is nine-coordinated. The separation in three m_J_ sublevels of the I(^5^D_0_ → ^7^F_1_) band does not rule out this hypothesis, because the separation is predicted also for the *D*_2h_ symmetry point group characteristic of a regular tricapped trigonal prism [57].

**Fig. 2:**
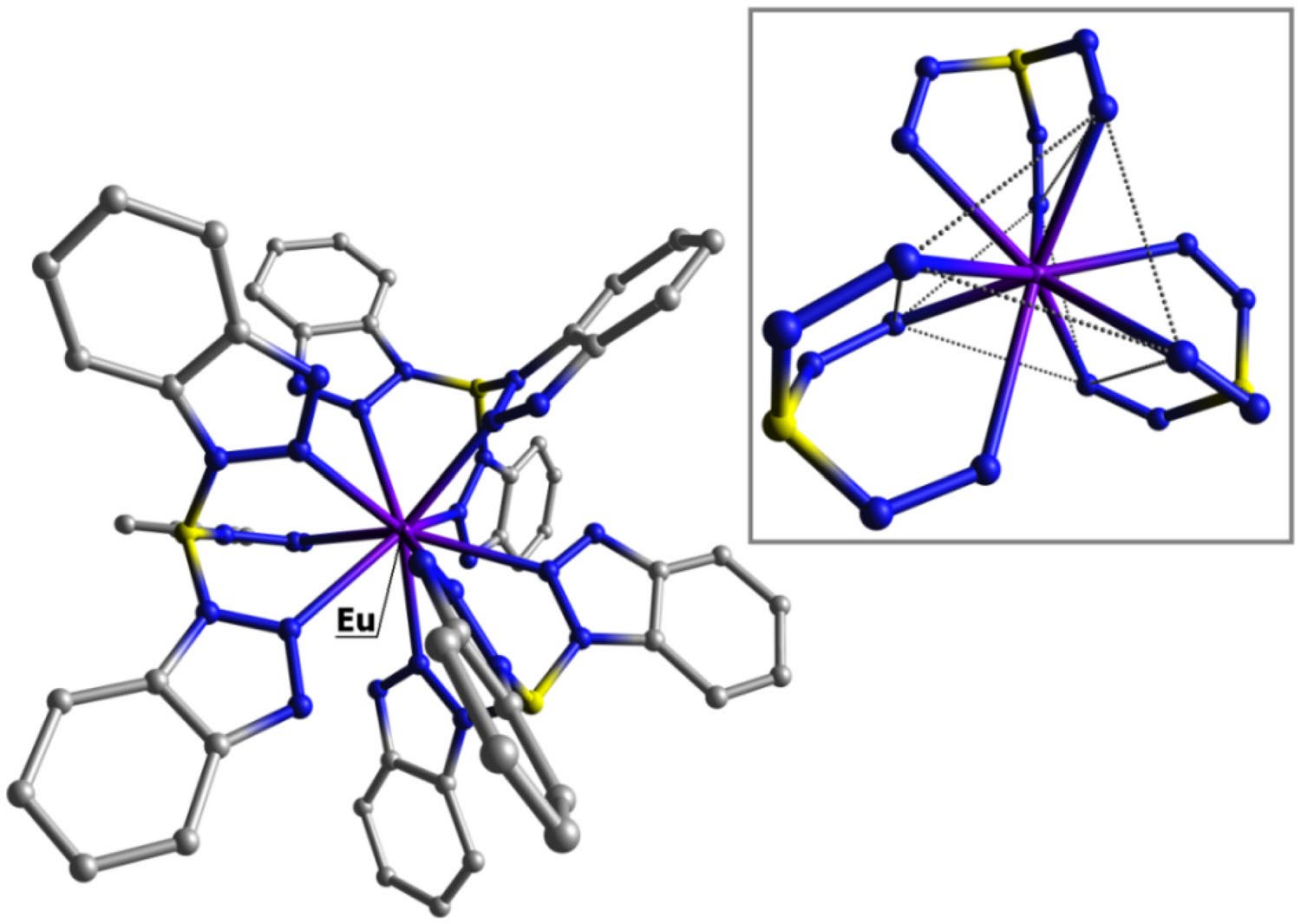
DFT-optimized geometry of [Eu(tbtz)_3_]. Hydrogen atoms are omitted for clarity. Inset: tricapped trigonal prism surrounding the Eu(III) centre. Colour map: Eu, violet; N, blue; C, grey; B, yellow. Selected computed Ln-N bond lengths (Á): [Eu(tbtz)_3_], 2.511 – 2.515 (trigonal prism), 2.729 – 2.742 (capped); [Gd(tbtz)_3_], 2.497 – 2.502 (trigonal prism), 2.735 – 2.739 (capped); [Tb(tbtz)_3_], 2.484 – 2.488 (trigonal prism), 2.720 – 2.736 (capped); [Y(tbtz)_3_], 2.493 – 2.497 (trigonal prism), 2.725 – 2.741 (capped)

Despite the fact that we were unable to obtain crystals suitable for X-Ray diffraction, the DFT-optimized geometry of [Eu(tbtz)_3_] confirms the proposed geometry for the complex, and the tricapped trigonal prism is predicted also for the first coordination sphere of analogous yttrium derivative, [Y(tbtz)_3_]. The computed ground-state geometry of [Eu(tbtz)_3_] is shown in Fig. 2. Selected computed bond lengths are collected in the caption of Fig. 2.

**Fig.3:**
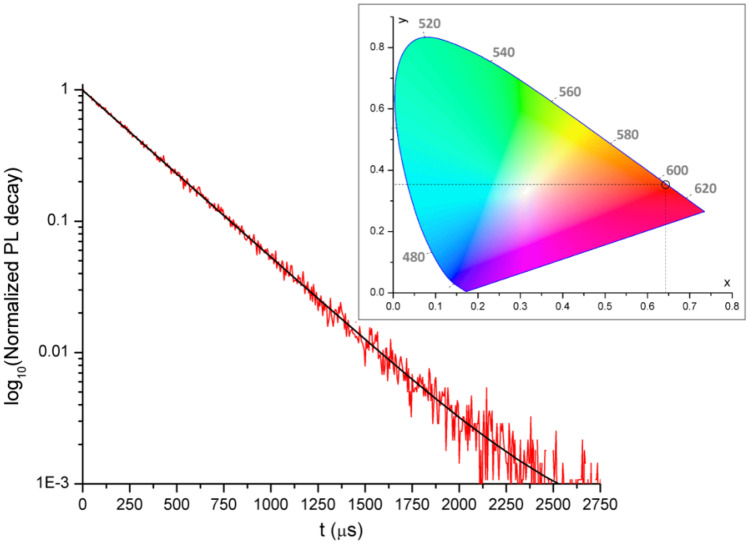
Semi-log plot of the luminescence decay curve of [Eu(tbtz)_3_] (red line) and monoexponential fit (black line). Solid sample, r.t., λ_excitation_ = 310 nm, λ_emission_ = 700 nm. Inset: CIE 1931 chromaticity diagram

The emission of [Eu(tbtz)_3_] falls in the reddish orange region of the CIE 1931 diagram with unitary colour purity, as shown in Fig. 3 [58]. The luminescence decay curve is monoexponential, as observable from the semi-log plot reported in Fig. 3. The measured lifetime value ($\tau$) is 0.341 ms. The intrinsic quantum yield ${Q }_{Eu}^{Eu}$ was estimated from the lifetime value on the basis of equation (1), where n indicates the refractive index of the sample. The value of 1.5 is assumed for solid state samples in this work fo comparative purposes [59], even if it is worth noting that refractive index may differ depending on the nature of the sample. I(^5^D_0_ → ^7^F_J_)/I(^5^D_0_ → ^7^F_1_) is the ratio between the total integrated emission from the Eu(^5^D_0_) level to the ^7^F_J_ manifold and the integrated intensity of the transition ^5^D_0_ → ^7^F_1_ [59].

1 $$Q_{Eu}^{Eu}=14.65\;n^3\frac{I({}^5D_0\rightarrow{}^7F_J)}{I({}^5D_0\rightarrow{}^7F_1)}\tau(s)$$

The ${Q }_{Eu}^{Eu}$ value for solid [Eu(tbtz)_3_] is 19%. The $\tau$ value is much lower than the reference value of 1.84 ms obtained for [Eu(Tp)_3_] under comparable conditions. As a consequence, also the intrinsic quantum yield is lower, being that of [Eu(Tp)_3_] around 43% [39]. Despite this limitation, the formal replacement of pyrazol-1-yl substituents with benzotriazol-1-yl broadens the absorption spectrum towards longer wavelengths and increases the absorption coefficients with respect to [Eu(Tp)_3_], making the complex bright photoluminescent. Luminescence data are collected in Table 1.

**Table 1:** Absorption and emission data of the complexes and of the doped polymers

Compound	^[a]^ UV–VIS, nm (ɛ, cm^−1^ M^−1^)	^[b]^ PL, nm	^[b]^ PLE, nm	^[b]^ $\tau$(ms)	${\mathbf{Q}}_{\mathbf{L}\mathbf{n}}^{\mathbf{L}\mathbf{n}}$(%)	CIE
[Y(tbtz)_3_]	278 (40,100)	–––––	–––––	–––––	–––––	–––––
[Eu(tbtz)_3_]	280 (37,400)	^[c]^ 579 (^5^D_0_ → ^7^F_0_, 0.8%) 588, 592, 596 (^5^D_0_ → ^7^F_1_, 8.8%) 613, 616 (^5^D_0_ → ^7^F_2_, 28.1%) 649, 651 (^5^D_0_ → ^7^F_3_, 2.4%) 699 (^5^D_0_ → ^7^F_4_, 59.9%)	^[d]^ $\le$350 (ligand excitation, max 309) 361, 375, 383 (Eu^3+^ excitation) 394 (^5^L_6_ ← ^7^F_0_ Eu^3+^ excitation)	^[c,d]^ 0.341	19	x = 0.643 y = 0.353
[Gd(tbtz)_3_]	276 (36,800)	^[e]^ 364 very weak	–––––	^[f,g]^ < 5·10^–4^	–––––	–––––
[Tb(tbtz)_3_]	274 (36,100)	^[c]^ 489 (^5^D_0_ → ^7^F_6_, 16.9%) 542 (^5^D_0_ → ^7^F_5_, 58.8%) 583, 587 (^5^D_0_ → ^7^F_4_, 14.1%) 620 (^5^D_0_ → ^7^F_3_, 10.2%)	^[h]^ $\le$343 (ligand excitation, max 307) 350, 367, 378 (Tb^3+^ excitation)	^[f,i]^ 0.383	9	x = 0.354 y = 0.566
[Eu(tbtz)_3_]@PMMA	–––––	^[c]^ 579 (^5^D_0_ → ^7^F_0_, 0.9%) 588, 594 (^5^D_0_ → ^7^F_1_, 11.7%) 612, 617 (^5^D_0_ → ^7^F_2_, 37.2%) 650 (^5^D_0_ → ^7^F_3_, 2.7%) 695, 700 (^5^D_0_ → ^7^F_4_, 47.5%)	^[d]^ $\le$350 (ligand excitation, max 309) 361 (Eu^3+^ excitation) 394 (^5^L_6_ ← ^7^F_0_ Eu^3+^ excitation)	^[c,d]^ 0.370	16	x = 0.646 y = 0.351
[Tb(tbtz)_3_]@PMMA	–––––	^[c]^ 489 (^5^D_0_ → ^7^F_6_, 18.2%) 544 (^5^D_0_ → ^7^F_5_, 56.7%) 583 (^5^D_0_ → ^7^F_4_, 15.2%) 621 (^5^D_0_ → ^7^F_3_, 9.9%)	^[h]^ $\le$350 (ligand excitation, max 311) 351, 368, 378 (Tb^3+^ excitation)	^[f,i]^ 0.630	13	x = 0.355 y = 0.571

^[a]^CH_2_Cl_2_ solution, 298 K

^[b]^ Solid sample, r.t

^[c]^ λ_excitation_ = 310 nm

^[d]^ λ_emission_ = 700 nm

^[e]^ λ_excitation_ = 280 nm

^[f]^ λ_excitation_ = 320 nm

^[g]^ λ_emission_ = 410 nm

^[h]^ λ_emission_ = 542 nm

^[i]^ λ_emission_ = 544 nm

**Fig. 4:**
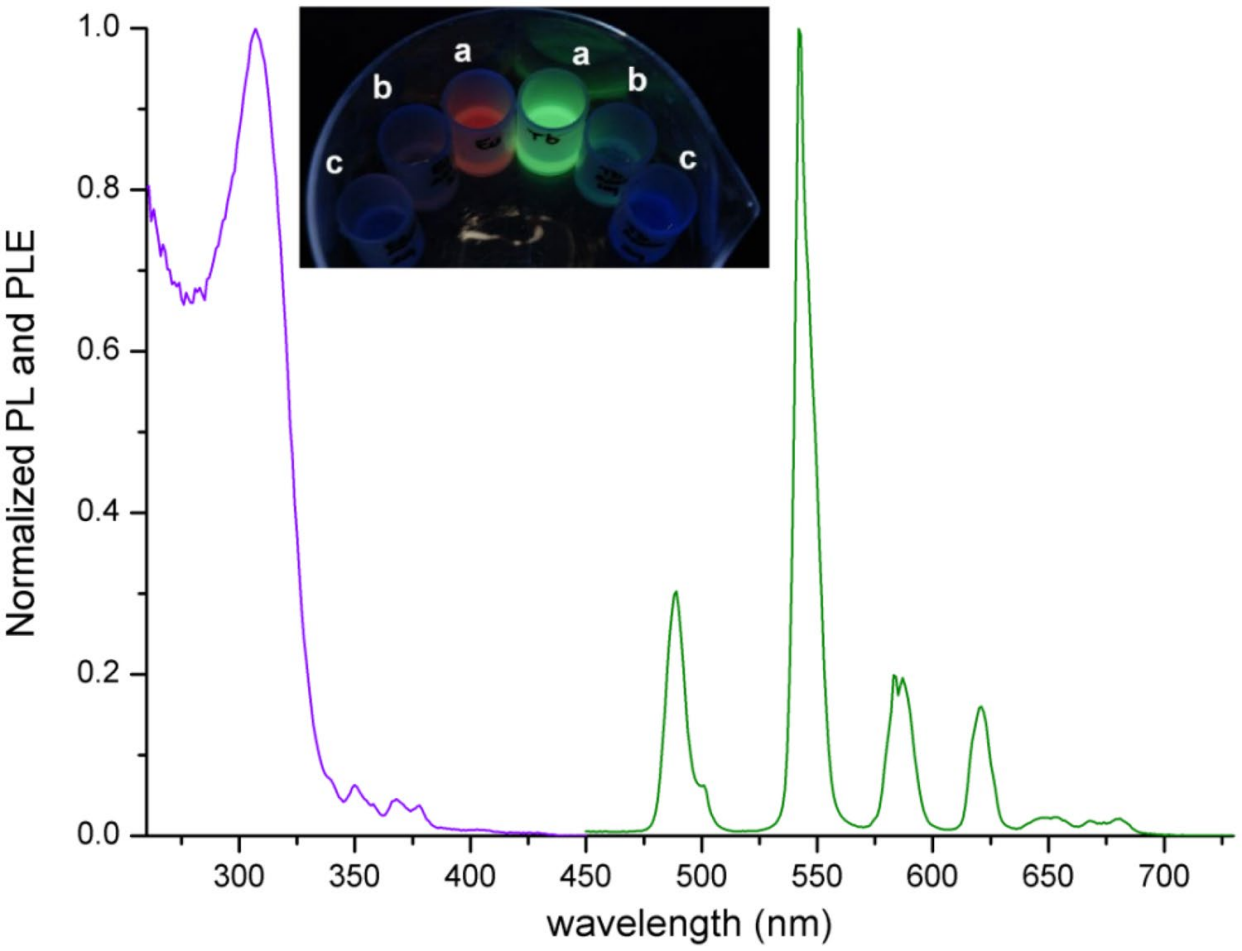
PL (green line, λ_excitation_ = 310 nm) and PLE (violet line, λ_emission_ = 542 nm) of solid [Tb(tbtz)_3_] at room temperature. Picture: emissions of 10^–4^ M CH_2_Cl_2_ solutions of [Ln(tbtz)_3_] (Ln = Eu, Tb) under UV light **a** and after addition of one **b** and two **c** equivalents of CH_3_SO_3_H

Another difference of [Eu(tbtz)_3_] with respect to [Eu(Tp)_3_] is that water suspensions of the last compound maintain good luminescence [41]. On the contrary, the luminescence of [Eu(tbtz)_3_] is easily quenched by traces of water and the compound is moisture-sensitive. Such a property can be tentatively ascribed to the presence of non-coordinating nitrogen atoms in the molecular structure (N3 in the benzotriazol-1-yl skeleton), that can interact with water through hydrogen bonds, thus favouring the vibrational relaxation of the excited state. The presence of free nitrogen atoms could also explain the immediate interaction of [Eu(tbtz)_3_] with acids in dichloromethane solution, causing the complete quench of the luminescence (see Fig. 4).

The terbium derivative [Tb(tbtz)_3_] shows appreciable green luminescence in dichloromethane solution and as solid sample upon excitation with UV light. DFT calculations predict a tricapped trigonal prismatic geometry strictly comparable to that obtained for the analogous europium complex. Computed data are summarized in the caption of Fig. 2. As for [Eu(tbtz)_3_], the luminescence is quenched by water and by addition of acids to dichloromethane solutions (see Fig. 4). The PL spectrum reported in Fig. 4 shows four bands attributable to the ^5^D_0_ → ^7^F_J_ (J = 6 – 3) transitions in the 450 – 650 nm range, together with weak signals at longer wavelengths related to lower J values. The most intense band falls at 542 nm and corresponds to the ^5^D_0_ → ^7^F_5_ transition. The PLE spectrum (Fig. 4) is comparable to that of [Eu(tbtz)_3_], with a strong contribution from the coordinated ligands for wavelengths below 350 nm and weak signals related to the direct excitation of Tb(III). The $\tau$ value obtained from the monoexponential fit of the luminescence decay curve is 0.383 ms. On admitting a Tb(III) radiative lifetime ($\tau$_rad_) of 4.75 ms [60], the intrinsic quantum yield ${\mathrm{Q}}_{\mathrm{T}\mathrm{b}}^{\mathrm{T}\mathrm{b}}$ is estimated on the basis of Eq. (2) around 9%. Photoluminescence data of [Tb(tbtz)_3_] are summarized in Table 1.

2 $${{Q}}_{{T}{b}}^{{T}{b}}=\frac{{\tau }({m}{s})}{4.75}$$

The synthesis of homoleptic tbtz complexes was extended to Gd(III), in order to obtain information about the excited states of the coordinated ligands from the PL spectrum of [Gd(tbtz)_3_]. Unfortunately, the phosphorescence of the compound is too low to be detected at room temperature, differently to what observed in the past for [Gd(Tp)_3_] [39]. Only a very weak band centred at 364 nm is observable, attributable to fluorescence from coordinated ligands on the basis of the fast luminescence decay [61].

**Fig. 5:**
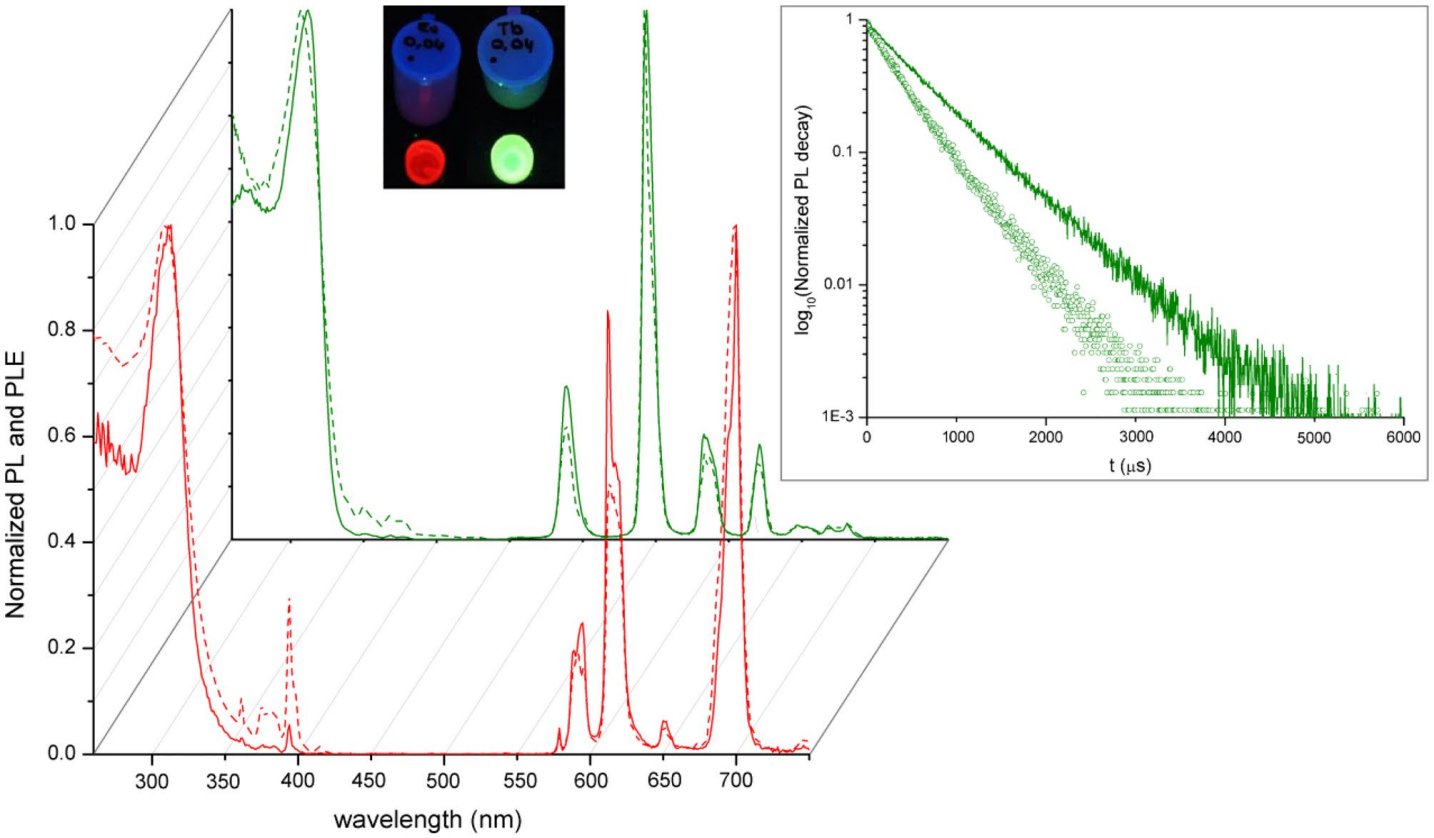
PL and PLE spectra of solid [Ln(tbtz)_3_] and [Ln(tbtz)_3_]@PMMA. Ln = Eu, red; Ln = Tb, green. Solid line, doped polymer; dashed line, pure complex. Inset: [Ln(tbtz)_3_]@PMMA samples (0.040 g_complex_/g_polymer_) under UV irradiation. Inset: semi-log plot of the luminescence decay curves of solid [Tb(tbtz)_3_]@PMMA (solid line) and [Tb(tbtz)_3_] (dots), λ_excitation_ = 320 nm, λ_emission_ = 542 nm

The appreciable photoluminescence of [Eu(tbtz)_3_] and [Tb(tbtz)_3_] prompted to study their use as dopants in plastic matrices, PMMA in particular. Highly photoluminescent materials were obtained with very low quantities of embedded complex, from 0.020 to 0.060 g_complex_/g_polymer_. The use of higher quantities of complex afforded materials with reduced transparency. Selected samples under UV light are shown in Fig. 5. The PL and PLE spectra, together with the time-resolved spectra, do not vary on changing the concentration of dopant. As observable in Fig. 5, the relative intensities of the ^5^D_0_ → ^7^F_J_ transitions in the PL spectrum of [Eu(tbtz)_3_]@PMMA are different with respect to the pure complex, suggesting that the PMMA chains alter the coordination sphere surrounding the Eu(III) centre. The most striking change is the increased intensity of the ^5^D_0_ → ^7^F_2_ transition, with a peak at 612 nm. It is however worth noting that the excitations related to the tbtz ligands in the PLE spectrum are unaffected by the PMMA matrix. This last consideration is maintained for [Tb(tbtz)_3_]@PMMA. The PL spectrum of the terbium-doped material is quite similar to that of the pure complex, but the meaningful variation of the luminescence decay curve (Fig. 5) highlights also in this case the interaction of the PMMA chains with the lanthanide complex. The $\tau$ value of [Tb(tbtz)_3_]@PMMA is 0.630 ms, longer than that of pure [Tb(tbtz)_3_], with a consequent increase of ${\mathrm{Q}}_{\mathrm{T}\mathrm{b}}^{\mathrm{T}\mathrm{b}}$, the estimated value being around 13%. The lifetime values of [Eu(tbtz)_3_] and [Eu(tbtz)_3_]@PMMA are instead similar, but the different PL spectra suggest that this outcome is merely a coincidence. The photoluminescence data of [Ln(tbtz)_3_]@PMMA materials are summarized in Table 1.

## Conclusion

In this paper we described the synthesis and photophysical characterization of luminescent europium and terbium complexes with a scorpionate ligand based on benzotriazole. The new compounds are rare examples of homoleptic tris(benzotriazol-1-yl)borate metal complexes [62]. [Eu(tbtz)_3_] maintained the peculiar emission features already observed for the analogous tris(pyrazol-1-yl)borate derivative, in particular the unusually high intensity of the ^5^D_0_ → ^7^F_4_ transition around 700 nm, in agreement with comparable first coordination spheres. The change of the azole in the scorpionate skeleton however caused a variation in the excitation range, in the luminescence lifetimes and in the quantum yields. Moreover, the tris(benzotriazol-1-yl)borate derivatives revealed to be more influenced by the surrounding environment with respect to tris(pyrazol-1-yl)borate homoleptic complexes, making these species of potential interest in the field of luminescent sensors. Despite the fact that bright luminescent materials were obtained by embedding the complexes in poly(methyl methacrylate), the photoluminescence data clearly indicated that the coordination sphere was altered by the polymer chains.

## Data Availability

The datasets generated during the current study are available from the corresponding author on reasonable request.
